# Impact of meteorological factors on influenza incidence in Wuxi from 2014 to 2019: a time series and comprehensive analysis

**DOI:** 10.3389/fpubh.2025.1656111

**Published:** 2025-08-08

**Authors:** Menglan He, Yan Wang, Xinliang Ding, Yumeng Gao, Chao Shi

**Affiliations:** ^1^School of Public Health, Nanjing Medical University, Nanjing, China; ^2^The Affiliated Wuxi Center for Disease Control and Prevention of Nanjing Medical University, Wuxi Center for Disease Control and Prevention, Wuxi, China

**Keywords:** influenza, meteorological factors, generalized additive model, time series study, meta-analysis

## Abstract

**Background:**

Although the association between meteorological factors and influenza was numerously documented, the results are inconsistent, requiring a meta-analysis for synthesis. A recent analysis of the association between influenza and meteorological factors was conducted in Wuxi, China.

**Methods:**

Meteorological data and laboratory-confirmed influenza cases from 2014 to 2019 were collected in Wuxi. The exposure-response relationship was analyzed using a generalized additive model. Then we performed subgroup analysis based on age and gender. Finally, meta-analysis was utilized to aggregate the total influence of meteorological factors on influenza.

**Results:**

A total of 5,306 influenza cases were reported. Seven influenza peaks, observed every winter to spring and only one summer (2015), were noted. For a unit increase in weekly average pressure, temperature, wind speed, relative humidity, precipitation, and sunshine duration, the risk of influenza increased by 7.37%, decreased by 8.39%, decreased by 33.83%, increased by 6.44% when relative humidity was >68.86%, increased by 19.91% when precipitation was ≤ 6.13 mm, and decreased by 11.41% when sunshine duration was ≤ 6.30 h, respectively. No significant gender differences were observed. The impacts of air pressure, temperature, precipitation, and sunshine duration on children aged 3–14 were greater than on other age groups. Compared with the meta-analysis, the pooled effect of ambient temperature was consistent. In subgroup and meta-regression analysis, significant differences were found in the children group.

**Conclusion:**

This study provides further insight into the effects of meteorological factors on influenza incidence, especially the impact on children, helping enhance the level of influenza monitoring and early warning research.

## 1 Introduction

Influenza is an acute respiratory infectious disease caused by the influenza virus, characterized by rapid onset and swift transmission. In China, it is classified as a Category C notifiable infectious disease (1). Since 2014, the H1N1 influenza virus, which caused the 2009 pandemic, has been reclassified from a Category B to a Category C disease (2). At the end of 2019, China revised its influenza diagnostic criteria (3). Seasonal influenza outbreaks result in a significant global burden of morbidity, severe cases, and mortality. The World Health Organization estimates that there are approximately 1 billion cases of influenza worldwide each year, including 3–5 million severe cases and 290–650 thousand deaths (4). Influenza also exerts a heavy toll in China, with reports indicating that between 2010 and 2015, influenza caused an average of 88,100 respiratory-related deaths annually, imposing a substantial economic burden (5). Despite the effectiveness of vaccination in preventing infection, low vaccination rates, due to various factors, continue to pose a persistent threat to global public health security (6, 7).

The survival ability of the influenza virus in the external environment is influenced by various meteorological factors, but research findings vary across different geographic regions. In temperate regions, influenza activity typically peaks during the cold winter months (7, 8). Studies from Finland and South Korea have shown that low temperatures and low humidity facilitate the transmission and survival of the influenza virus (9, 10). In contrast, the seasonality of influenza is less distinct in tropical and subtropical regions. A study in Wuhan found that when the average temperature exceeds a certain threshold, the risk of influenza incidence is significantly positively correlated with temperature, peaking at higher temperatures (11). Additionally, other meteorological factors such as precipitation, wind speed, and sunshine duration have also been associated with influenza incidence (12). However, the seasonal patterns of influenza and the predominant circulating strains have shifted during the COVID-19 pandemic. To our knowledge, no specific studies have yet explored the relationship between meteorological factors and influenza risk post-pandemic. Most existing epidemiological studies are limited to time-series analyses in individual cities, lacking a systematic assessment of regional heterogeneity. This inconsistency in findings not only hinders a comprehensive understanding of the role of meteorological factors in influenza incidence but also provides insufficient evidence for public health decision-making. Therefore, further multi-regional and systematic research is essential to explore the relationship between meteorological factors and influenza incidence, as well as regional differences, which is crucial for developing effective influenza prevention and control strategies.

Wuxi, an important economic hub in the Yangtze River Delta, is situated on the shores of Taihu Lake in southeastern Jiangsu Province. With a resident population of approximately 7.46 million based on 2024 Statistical Yearbook, it features a subtropical monsoon climate characterized by distinct seasons, abundant rainfall, and ample sunshine (13, 14). Leveraging its citywide healthcare information system, Wuxi has developed a comprehensive influenza surveillance data platform, offering reliable data to support research into the correlation between influenza incidence and meteorological factors. This study aims to systematically investigate the relationship between meteorological factors and influenza risk, as well as their regional heterogeneity, through a two-stage analysis. In the first stage, time series analysis of meteorological and influenza case data from Wuxi City (2014–2019) will be conducted to determine the exposure-response relationship at the optimal lag week, assess the impact of meteorological changes on influenza risk, and identify regional epidemic characteristics. In the second stage, the results from the first stage will be combined with other studies for a meta-analysis to evaluate the overall effect of meteorological factors on influenza incidence risk and its heterogeneity across regions.

## 2 Methods

### 2.1 Study site and data collection

Wuxi (31°07′-32°02′N, 119°31′-120°36′E) in the eastern part of Jiangsu Province, China, lies within the subtropical humid climate zone, characterized by distinct four seasons (13). Daily laboratory-confirmed influenza cases between January 1, 2014, and December 31, 2019 were obtained from the database of the National Center for Public Health Surveillance and Information Services, China Information System for Disease Control and Prevention (CISDCP) (15). The screening criteria were the current address in Wuxi and the date according to the onset date of the case. Laboratory-confirmed influenza cases were diagnosed based on the diagnostic criteria outlined by the National Health Commission of the People’s Republic of China (WS 285–2008) (16). The cases were reported online via the database within 24 h of diagnosis by using a standardized form including demographic information (gender, date of birth, and current address) and case description (date of symptoms onset).

Daily meteorological data for this study was collected from Wuxi Municipal Meteorological Monitoring Center. The meteorological data included the daily averages for atmospheric pressure, temperature, relative humidity, wind speed, precipitation, and sunshine duration. The daily average values of meteorological factors are calculated based on the average values of 2 am, 8 am, 2 pm, and 8 pm for each day at a single fixed-site station in Xishan district of Wuxi. Meteorological data and laboratory-confirmed influenza cases were aggregated into weekly averages, with the week beginning on January 1, 2014. Each week is calculated by 7 days, and the data are averaged for each 7-day period, for use in subsequent modeling analyses.

### 2.2 Search strategy and study selection

A systematic literature search strategy was performed for articles from January 1, 2000, to February 27, 2024, which shows the association between meteorological factors and the risk of influenza. The China National Knowledge Infrastructure (CNKI), Wanfang Database, VIP Database, PubMed, Web of Science, Embase, and Cochrane Library were searched with the combination of terms “influenza” and “meteorological factors.” We conducted a data retrieval update in January 2025 to include relevant studies that may have been published shortly before the review’s completion. This review was carried out following PRISMA and registered with PROSPERO (CRD42021284952), and the detailed information was provided in Supplementary File.

Studies focusing on the association of meteorological factors with the risk of influenza, which met the following conditions, were included: (1) Studies report laboratory-confirmed influenza cases (excluding influenza-like illness), with outcome variables in monthly, weekly, or daily cases; (2) Exposure variables include environmental factors such as temperature, relative humidity, precipitation, atmospheric pressure, wind speed, and sunshine duration; and (3) Studies provide effect estimates (e.g., relative risk (RR), odds ratio (OR) with 95% confidence intervals (95% CI), or other estimates convertible to RR and 95% CI).

### 2.3 Data extraction

To extract the effect, estimate and its 95% CI and calculate these estimates based on a one-unit increase in each meteorological factor. Alternatively, converted them into standardized relative risk (RR) using the following Equation (1) (17):


(1) $${\mathrm{RR}}_{\text{standardized}}={\mathrm{RR}}_{\text{Original}}^{\text{Increment}(1)/\text{Increment}(\text{Original})}$$

If a study reported excess risk (ER), this value can be converted using the following Equation (2):


(2) RR=ER+1

When extracting effect sizes, prioritize the largest effect size if both single-day and cumulative lags are reported. For multi-center studies, use the overall effect size or combine results from multiple regions. If different influenza subtypes are reported, include data for each subtype separately. Age-stratified results should provide effect sizes for children aged ≤18 years to facilitate subsequent subgroup analysis. In studies with various association curves (e.g., U-shaped, J-shaped, V-shaped), the standardized RR is calculated by comparing meteorological factors at different percentiles to the reference value (RR = 1). For cold effects, RR is calculated by comparing the 1st to 25th percentiles of temperature with the reference value, and for hot effects, the 75th to 99th percentiles are used. For linear associations, the effect estimate provided by the authors is used, assuming it relates to the average meteorological factor. The conversion for other meteorological factors follows a similar approach. Literature screening and data extraction are independently performed by two researchers, with a third-party opinion sought in case of disagreement.

### 2.4 Statistical analysis

In the time series study, the Generalized Additive Model (GAM) with a quasi-Poisson regression link function was used to assess the effect of meteorological factors on influenza incidence. Given that previous studies indicate a delayed impact of meteorological factors on influenza risk, a maximum lag time of 0–2 weeks was set to evaluate the association. The specific model is shown in Equation (3).


(3) $$\begin{array}{l} \mathrm{Log}E(Y_{t})=\beta +s(x,\mathrm{df})+s(\text{week},\mathrm{df})+\mathrm{Log}E(Y_{t-1})+\\ \text{season}+\text{holiday} \end{array}$$

In the model, *Y_t_* represents the number of influenza cases in week; *β* denotes the intercept of the entire model; *x* refers to the meteorological factors to be analyzed; *week* is used to control for time trends; *Y_t − 1_* represents the influenza cases from the previous week to control for short-term autocorrelation in the incidence series; *holiday* indicates the number of holidays (including weekends and public holidays) in the given week; and *season* accounts for seasonal factors to control for seasonal fluctuations in influenza incidence. *s*() represents the thin plate spline function. The optimal degrees of freedom for the spline function were determined using the Generalized Cross-Validation (GCV) score, with the final degrees of freedom for meteorological factors set to 2 and for time trends set to 6. The GCV scores of models with different lag weeks were compared, and the lag week corresponding to the lowest score was chosen as the optimal lag period. The exposure-response curve of meteorological factors and influenza incidence risk was plotted based on the best lag period. If the curve is linear or approximately linear, the effect of meteorological factors on influenza was estimated directly; otherwise, a piecewise linear regression was used for estimation. In addition, subgroup analysis was conducted to assess the robustness of the association, with inter-group heterogeneity tested using Cochran’s Q test.

In the meta-analysis, effect estimates and 95% CI from the included studies were combined with those from the first-stage analysis. Heterogeneity was assessed using the I^2^ statistic, with a random-effects model applied if I^2^ > 50% and *p* < 0.01, otherwise a fixed-effects model was used. Additionally, in the presence of significant heterogeneity, subgroup analyses and meta-regression were performed to explore potential sources of heterogeneity. Sensitivity analysis was conducted by sequentially excluding individual studies to assess their impact on the overall estimate. Funnel plots were generated, and Egger’s and Begg’s tests were employed to analyze potential publication bias. All statistical tests for the meta-analysis were performed using R 4.3.1 with the “meta” package. The significance level was set at *α* = 0.05.

## 3 Results

### 3.1 Descriptive analysis

Table 1 described the demographic and meteorological information on laboratory-confirmed influenza cases. A total of 5,306 confirmed influenza cases were reported in Wuxi. The time series distribution of weekly influenza cases and meteorological factors was shown in Supplementary Figure 1. Influenza cases exhibited cyclical variation, with seven peaks observed during the study period (2014–2019). These peaks occurred every winter to the following spring, with one exception in the summer of 2015.

**Table 1:** Description of weekly influenza cases and meteorological factors in Wuxi, 2014–2019.

Variable	*n* (%)	Mean (SD)	Min	P25	Median	P75	Max
Cases	5,306	16.95 (54.19)	0.00	1.00	5.00	16.00	745.00
Seasons							
Spring	719 (13.55)	9.1 (9.69)	0.00	1.00	6.00	15.50	50.00
Summer	380 (7.16)	4.81 (6.93)	0.00	0.00	2.00	6.00	33.00
Autumn	374 (7.05)	4.79 (7.44)	0.00	0.00	2.00	5.75	46.00
Winter	3,833 (72.24)	49.78 (101.94)	1.00	10.00	24.00	54.00	745.00
Sex groups							
Male	2,728 (51.41)	8.72 (28.49)	0.00	0.00	3.00	8.00	389.00
Female	2,578 (48.59)	8.24 (25.82)	0.00	0.00	2.00	8.00	356.00
Age groups							
0–2 years	502 (9.46)	1.60 (3.26)	0.00	0.00	1.00	2.00	37.00
3–14 years	2,839 (53.51)	9.07 (43.67)	0.00	0.00	2.00	6.00	632.00
15–59 years	1,448 (27.29)	4.63 (8.86)	0.00	0.00	2.00	5.00	69.00
≥60 years	517 (9.74)	1.65 (5.05)	0.00	0.00	0.00	1.00	49.00
Meteorological factors							
Pressure (hPa)		1016.18 (8.67)	997.76	1007.90	1017.11	1023.30	1035.43
Temperature (°C)		17.28 (8.70)	−0.97	9.59	18.14	24.36	35.13
Relative humidity (%)		73.14 (8.98)	50.29	66.57	73.71	79.71	94.57
Precipitation (mm)		3.57 (5.47)	0.00	0.27	1.69	4.20	42.39
Wind speed (m/s)		2.17 (0.45)	1.19	1.84	2.11	2.43	3.87
Sunshine duration (h)		4.75 (2.47)	0.00	2.83	4.79	6.66	12.13

Supplementary Table 1 demonstrated the spearman correlation between weekly influenza cases and meteorological factors. The weekly mean temperature was significantly and negatively correlated with weekly mean pressure. The weekly relative humidity was significantly and negatively weekly mean sunshine duration, and significantly and positively correlated with weekly mean precipitation.

### 3.2 Association between meteorological factors on weekly laboratory-confirmed influenza cases

**Figure 1:**
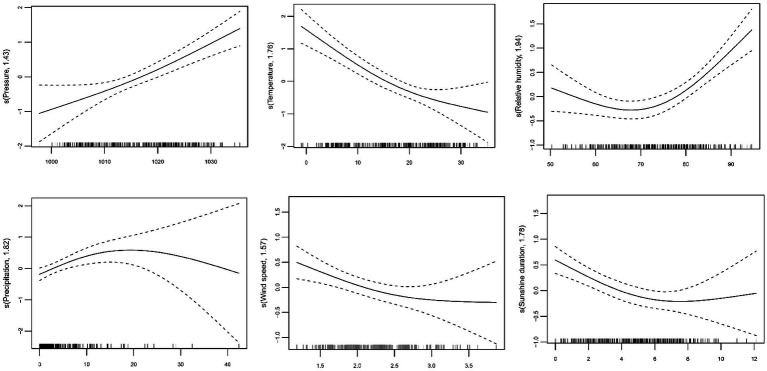
The exposure-response curves between meteorological factors and influenza.

According to the GCV score, the models with the optimal lag weeks for the different variables were established (Supplementary Table 2). Figure 1 showed the exposure-response curve between the weekly meteorological levels and influenza cases. We used linear terms instead of smoothing terms to assess the impact of average atmospheric pressure, average temperature, and average wind speed on influenza incidence risk. For each 1 hPa increase in average atmospheric pressure, the influenza incidence risk increased by 7.37% (95% CI: 4.65 to 10.17%). On the contrary, a 1°C increase in average temperature led to an 8.39% decrease (95% CI: −10.98% to −5.73%), and a 1 m/s increase in average wind speed resulted in a 33.83% reduction (95% CI: −48.79% to −14.50%). Subgroup analysis showed similar trends across sex and age groups (Table 2). Interestingly, we found that the effects of atmospheric pressure (Q = 11.32, *p* = 0.0101) and temperature (Q = 10.14, *p* = 0.0174) in children aged 3–14 years were significantly higher than other age groups (Supplementary Table 3). The exposure-response curves for relative humidity, precipitation, and sunshine duration showed non-linear effects (Figure 1). When relative humidity >68.86%, each 1% increase in relative humidity was associated with a 6.44% increase (95% CI: 3.76 to 9.14%). Subgroup analysis by sex and age showed consistent trends in the effect of relative humidity. Further heterogeneity test analysis revealed no significant differences between sexes (Q = 0.03, *p* = 0.8786) or age groups (Q = 3.15, *p* = 0.3687). When precipitation ≤ 6.13 mm, each 1 mm increase was associated with a 19.90% increase (95% CI: 12.40 to 27.92%). The exposure-response curve for sunshine duration indicated that when sunshine duration ≤ 6.30 h, each 1 h’s increase was associated with an 11.41% reduction (95% CI: −17.38% to −5.00%). Subgroup analysis showed similar effects of precipitation and sunshine duration as the overall results. Heterogeneity test revealed significant differences between age groups for both precipitation and sunshine duration, with a more pronounced impact in children aged 3–14 years (Table 3 and Supplementary Table 3).

**Table 2:** Impact of each unit increase in meteorological factors on influenza incidence risk and 95% CI (%).

Variable	Pressure	Temperature	Wind speed
Total	7.37 (4.65 to 10.17)^*^	−8.39 (−10.98 to −5.73)*	−33.83 (−48.79 to −14.50)*
Sex groups			
Male	7.20 (4.41 to 10.07)*	−8.21 (−10.88 to −5.45)*	−34.05 (−49.38 to −14.07)*
Female	7.52 (4.63 to 10.48)*	−8.50 (−11.23 to −5.69)*	−33.43 (−49.05 to −13.01)*
Age groups			
0–2 years	3.61 (0.22 to 7.10)*	−4.77 (−8.08 to −1.33)*	−27.68 (−47.53 to −0.34)*
3–14 years	11.11 (7.45 to 14.58)*	−10.43 (−13.45 to −7.31)*	−40.29 (−56.51 to −18.04)*
15–59 years	4.32 (1.55 to 7.16)*	−4.19 (−6.84 to −1.46)*	−25.73 (−42.92 to −3.30)*
≥60 years	4.86 (0.50 to 9.42)*	−6.79 (−11.20 to −2.17)*	−25.54 (−49.86 to 10.58)

*p < 0.05.

**Table 3:** Impact of each unit increase in meteorological factors on influenza incidence risk and 95% CI (%).

Variable	Relative humidity		Precipitation		Sunshine duration	
	≤68.86%	>68.86%	≤6.13 mm	>6.13 mm	≤6.30 h	>6.30 h
Totol	−3.39 (−6.76 to 0.10)	6.44 (3.76 to 9.14)*	19.91 (12.40 to 27.92)*	−0.75 (−4.74 to 3.41)	−11.41 (−17.38 to −5.00)*	8.21 (−7.60 to 26.71)
Sex groups						
Male	−2.98 (−6.64 to 0.83)	6.78 (4.07 to 9.56)*	21.38 (13.50 to 29.80)*	0.32 (−3.25 to 4.02)	−11.47 (−17.46 to −5.05)*	11.35 (−5.92 to 31.78)
Female	−2.87 (−6.43 to 0.83)	6.46 (3.67 to 9.33)*	18.34 (10.62 to 26.59)*	−2.09 (−7.07 to 3.17)	−11.35 (−17.67 to −0.05)*	3.58 (−14.10 to 24.90)
Age groups						
0–2 years	0.75 (−4.17 to 6.03)	6.05 (3.14 to 9.05)*	9.96 (3.53 to 16.79)*	−2.25 (−6.58 to 2.28)	−9.35 (−16.47 to −1.62)*	10.82 (−11.75 to 39.17)
3–14 years	−3.07 (−8.01 to 2.15)	8.19 (4.63 to 11.86)*	32.38 (22.85 to 42.87)*	−2.30 (−8.35 to 4.15)	−16.97 (−24.30 to −8.94)*	−9.76 (−30.71 to 17.53)
15–59 years	0.04 (−2.93 to 3.09)	5.16 (2.71 to 7.66)*	7.25 (0.01 to 15.01)*	−0.98 (−5.06 to 3.28)	−4.23 (−10.76 to 2.78)	1.84 (−15.50 to 22.73)
≥60 years	−5.67 (−10.79 to −0.03)*	3.71 (0.06 to 7.52)*	−9.68 (−20.84 to 3.41)	3.45 (−1.31 to 8.48)	11.47 (0.59 to 23.53)*	20.36 (−9.51 to 60.08)

*P < 0.05.

### 3.3 Quantitative data synthesis

**Figure 2:**
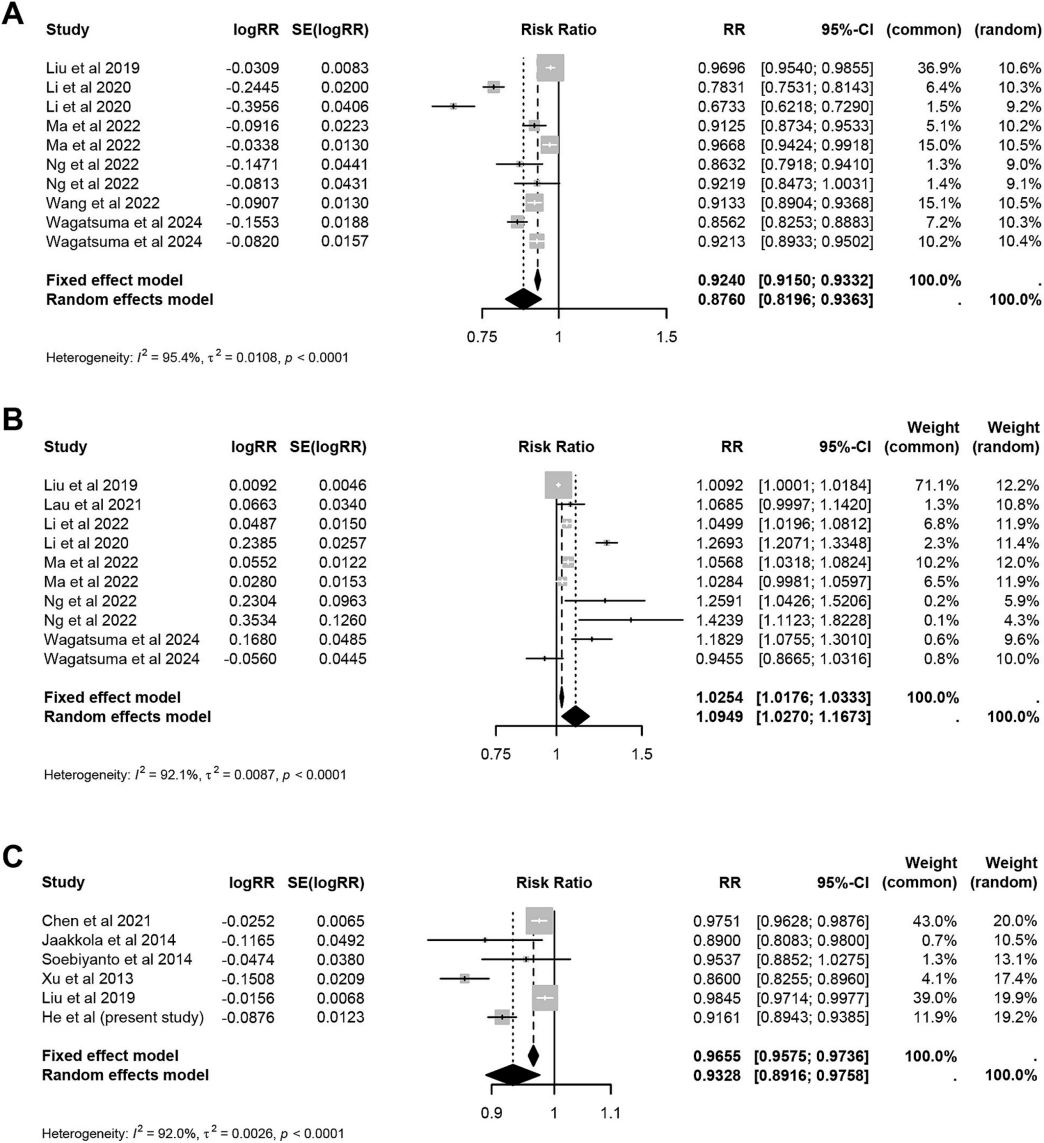
Meta-analysis of hot effect and cold effect of ambient temperature and risk of influenza. **(A)** Cold effect. **(B)** Hot effect. **(C)** Average effect.

**Figure 3:**
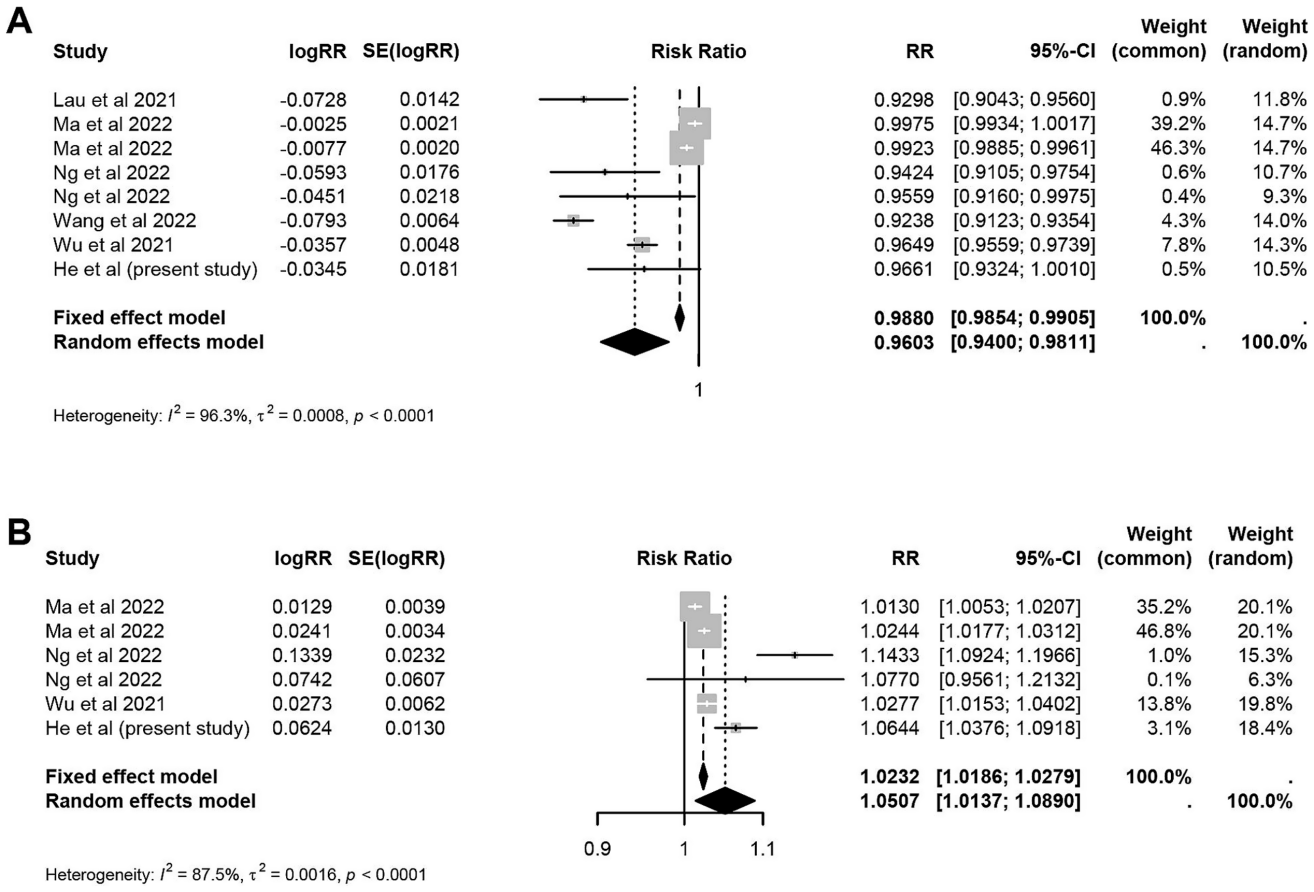
Meta-analysis of wet effect and dry effect of relative humidity and risk of influenza. **(A)** Dry effect. **(B)** Wet effect.

A total of 14 eligible papers were finally included (Supplementary Figure 2 and Supplementary Table 4). The meta-analysis indicated a significant statistical association between the unit increase in temperature and relative humidity and the risk of influenza. Specifically, for every 1°C increase in temperature under the cold effect, the risk decreased by 12.40% (RR = 0.88, 95% CI: 0.82 to 0.94). Conversely, the risk increased by 9.49% (RR = 1.09, 95% CI: 1.03 to 1.17) under the hot effect. The risk decreased by 6.72% for per 1°C increase in average temperature in terms of the linear associations (RR = 0.93, 95% CI: 0.89 to 0.98) (Figure 2). For relative humidity, the risk decreased by 3.97% (RR = 0.96, 95% CI: 0.94 to 0.98) for each 1% increase under dry effect. Conversely, under wet effect, the risk increased by 5.07% (RR = 1.05, 95% CI: 1.01 to 1.09) (Figure 3). Among other meteorological factors, only the combined effects of low wind speed and high precipitation showed a significant statistical association with influenza risk. Specifically, for every 1 m/s increase in low wind speed, the risk decreases by 19.69%. Conversely, for every 1 mm increase in high precipitation, the risk increased by 2.00% (Table 4). Further subgroup analysis indicated that the effects of temperature on influenza risk were significantly greater in children than in the overall population. Under cold effect, a 1°C increase in temperature reduced the influenza risk in children by 27.09% (RR = 0.73, 95% CI: 0.63 to 0.85). Under hot effect, a 1°C increase raised the risk by 26.93% (RR = 1.27, 95% CI: 1.21 to 1.33). The impact of temperature varied by climate zone, with subtropical regions showing stronger effects compared to temperate regions, where the hot effect did not have a statistically significant impact (Supplementary Table 5). The impact of relative humidity on different populations showed minimal differences, however, the dry effect was stronger in temperate regions than in subtropical regions (Supplementary Table 6).

**Table 4:** Meta-analysis of the risk of influenza with air pressure, sunshine duration, precipitation and wind speed.

Variables	Effect	*n*	Pooled RR(95%CI)	Heterogeneity	
				*I^2^*%	*p*-value
Air pressure	Mean effect	1	1.0737 (1.0465–1.1017)*	NA	NA
Sunshine duration	Low effect	1	0.8859 (0.8262–0.9500)*	NA	NA
	High effect	1	1.0820 (0.9240–1.2671)	NA	NA
Wind speed	Low effect	2	0.8031 (0.7142–0.9031)*	67.6%	0.0789
	Mean effect	1	0.6617 (0.5121–1.8550)	NA	NA
Precipitation	Low effect	1	1.1991 (1.1240–1.2792)*	NA	NA
	High effect	2	1.0200 (1.0108–1.0292)*	44.2%	0.1087
	Cumulative effect	1	1.0200 (0.9824–1.0592)	NA	NA

NA, Not Available; *P < 0.05.

Meta-regression analysis indicated that the study population significantly influenced the association between temperature and influenza risk, while regional climate affected the relationship between the dry effect of relative humidity and influenza risk (Supplementary Table 7). Moreover, sensitivity analysis demonstrated that excluding any single study did not significantly alter the overall effect size, indicating the robustness of the findings. However, publication bias analysis suggested a potential bias regarding the cold and hot effects of temperature (Supplementary Figure 3 and Supplementary Table 8).

## 4 Discussion

This study analyzed the association between meteorological factors and the risk of influenza incidence in Wuxi from 2014 to 2019, and conducted a meta-analysis incorporating comprehensive studies. A substantial number of studies indicated a close relationship between temperature and the risk of influenza incidence (18, 19). The results from Wuxi showed a negative linear relationship between temperature and influenza risk. Another study from Shaoyang, which is also located in a subtropical region, explored the association between meteorological factors and influenza using the GAM model (20). Consistent with our findings, a decrease in temperature was associated with an increased risk of influenza, which was further confirmed in the meta-analysis. A previous study suggested that during cold effect, we tend to congregate in poorly ventilated indoor spaces, which increases the risk of virus transmission (21). Low temperature, dry environments not only impair the respiratory mucosal barrier and reduce immune function but also enhance the survival capacity of the influenza virus, prolonging its persistence on surfaces or in the air (22, 23). Moreover, studies have observed a non-linear relationship between temperature and influenza risk. For example, research conducted in Shanghai and Macau revealed a U-shaped association between temperature and influenza cases, whereas a study in Shenzhen discovered an M-shaped relationship (24, 25). Beyond differences in statistical model settings, the heterogeneity of the association curves may be attributed to variations in geographical and climatic characteristics, such as latitude, solar radiation, and vitamin D levels (26, 27). Influenza cases in Wuxi are predominantly concentrated among children under 14, consistent with findings from other studies (16, 28). In the age subgroup analysis, we found that temperature has a greater impact on children, a finding also confirmed by the meta-analysis. This may be due to higher population densities in nurseries and schools, relatively weak hygiene awareness among children, and their underdeveloped immune system (29). This finding highlights the need for targeted prevention and control measures in collective settings such as schools. Given that children are a high-risk group for influenza, interventions like improving ventilation, promoting seasonal flu vaccinations, and encouraging good hygiene practices are crucial for reducing the risk of influenza among children in these settings. The impact of low temperatures on influenza is well-established. However, a small peak in influenza cases was observed in Wuxi during the summer of 2015, a pattern that was also reported in cities like Hangzhou and Shenzhen (30, 31). This phenomenon may be attributed to reduced outdoor activities and a tendency to stay indoors in high-temperature environments. The widespread use of air conditioning in such settings leads to poor ventilation, creating favorable conditions for viral transmission and cross-infection.

This study found that influenza cases in Wuxi exhibited a U-shaped correlation with relative humidity, achieving statistical significance at relative humidity levels above 68.86%. A similar time-series analysis in Shenzhen confirmed this association (31). A multi-region modeling analysis indicated that in subtropical and tropical regions, high relative humidity is linked to with increased influenza activity (32). One study mentioned that droplets produced during coughing or sneezing expand in humid air, which allows them to be more effectively retained in the respiratory tract (33). This phenomenon may explain the observed increase in influenza incidence in high humidity environments. Our meta-analysis further also confirmed that both low and high relative humidity are conducive to influenza incidence. Lowen et al. (34) guinea pig experimental study demonstrated that the stability of the influenza virus is primarily influenced by temperature and relative humidity. Specifically, the virus is most stable at low relative humidity, least stable at moderate relative humidity, and relatively stable at high relative humidity. In the subgroup analysis, low relative humidity had a greater impact on influenza incidence in temperate regions compared to subtropical regions, which aligns with higher influenza incidence in some areas during summer (10, 31). The occurrence of influenza under “cold and dry” and “humid and rainy” climate conditions may be closely related to the U-shaped relationship between influenza activity and relative humidity. This indicates that increased influenza activity under both extremely low and high humidity conditions is a key factor driving this phenomenon.

Research on the association between influenza and the other four meteorological factors is limited. However, some studies indicated that high atmospheric pressure is associated with an increase in influenza cases in Guangzhou, China, and Maricopa County, USA. These findings align with those observed in Wuxi (35, 36). One hypothesis suggests that high pressure typically indicates sunny weather, leading to increased outdoor activities, which subsequently raises the risk of viral transmission (37). In Wuxi, precipitation is mainly observed in lower ranges, and when it is ≤6.13 mm, it shows positive correlation with influenza incidence. This finding aligns with studies from tropical regions like Hong Kong and Singapore (38, 39). In our meta-analysis, we found that high precipitation increases the risk of influenza incidence. Soebiyanto et al. (35) suggested that the impact of precipitation on influenza outbreaks was not due to changes in virus viability or host susceptibility, but rather to changes in host social behavior. Specifically, precipitation may lead individuals to spend more time indoors, thus increasing opportunities for interpersonal contact. This behavioral shift can, to some extent, facilitate viral transmission. The results from Wuxi indicated an approximately negative linear relationship between average wind speed and influenza cases. However, in our meta-analysis, this association was observed only at lower wind speeds. While some studies suggest that stronger winds help disperse aerosols in the air, thereby increasing the risk of influenza and other respiratory viral infections, relatively stagnant air conditions may allow influenza viruses to persist in localized environments. This persistence can lead to higher incidence rates in areas such as schools, daycare centers, or public places (40, 41). Research has shown that shorter sunshine duration is associated with an increased risk of influenza, consistent with findings from Nordic regions and Macau (25, 42). An experimental study simulating the effect of sunshine duration on influenza virus inactivation in aerosols observed a negative correlation between sunshine duration and the virus (43). One hypothesis is that reduced sunlight exposure decreases the synthesis of melatonin and vitamin D, thus impairing immune function. However, a study in Xiamen found that higher sunshine duration was associated with increased influenza risk in children (44), indicating that the mechanisms involved may vary by region or population. The exact reasons behind these observations remain unclear and require further investigation.

To our knowledge, this is the first study to integrate the GAM with meta-analysis to explore the association between meteorological factors and influenza risk, offering a more innovative approach that surpassed traditional meta-analytic methods. However, there were still limitations. First, the reported rate of asymptomatic influenza infection was 19.1% (45), however, influenza cases were based on surveillance data rather than all actual infections, which may have led to a misestimation of the association between meteorological factors and influenza risk. Second, the meteorological data used in this study was from a single fixed station in Wuxi, which may not accurately reflect the specific meteorological conditions experienced by each case. This limitation means that the individual exposure levels could not be precisely determined, and therefore, the findings may not directly apply to inferences at the individual level. Additionally, as an ecological study, some degree of ecological fallacy is inevitable. The incidence of influenza is also influenced by indoor temperature, living and working conditions, socio-economic status, and health behaviors, but these factors were not accounted for due to data collection limitations, potentially affecting the results.

In summary, this study emphasizes the critical role of combining meteorological surveillance and early warning systems in reducing the risk of influenza. We recommend the development of an integrated early warning system for seasonal influenza that accounts for regional climatic characteristics and incorporates short-term meteorological indicators such as temperature and relative humidity. This approach enhances the sensitivity of monitoring systems, enabling the early detection of potential outbreaks and facilitating timely interventions, such as issuing public health alerts. This proactive strategy is crucial for protecting vulnerable populations, such as children in schools. By applying our research findings to influenza risk assessment, we can predict the trends of influenza outbreaks, guide the formulation of preventive and control measures, and optimize the allocation of medical resources. Establishing an early-warning mechanism for influenza outbreaks not only reduces the incidence, severity, and mortality of influenza but also enhances public health preparedness and response capabilities.

## 5 Conclusion

This study aims to systematically analysis the association between meteorological factors and influenza risk through a two-stage analysis. The current study provides additional insights into how average pressure, temperature, wind speed, relative humidity, precipitation, and sunshine duration influence the risk of influenza, with particular attention to the impacts on different age groups. Understanding these factors is essential and valuable for enhancing influenza surveillance and early warning mechanisms.

## Data Availability

The data analyzed in this study is subject to the following licenses/restrictions: the dataset must remain anonymized, and any personally identifiable information should not be disclosed or used in any form. Requests to access these datasets should be directed to CS: wxcdcshichao@126.com.
