# Supplementary material for: Impact of meteorological factors on influenza incidence in Wuxi from 2014 to 2019: a time series and comprehensive analysis
Source: Front Public Health. 2025 Aug 8;13:1656111. doi: 10.3389/fpubh.2025.1656111 (PMC12370636; doi:10.3389/fpubh.2025.1656111)
Supplement: Supplementary file 1 [file Data_Sheet_1.docx]

Supplementary Material

# Search strategy

# Quality assessment

# Study characteristics

# Supplementary Tables

**Supplementary Table 1.** Spearman correlation between influenza cases and meteorological factors in Wuxi, 2014-2019.

**Supplementary Table 2.** GCV Scores of Meteorological Factors and Influenza Incidence Risk Models for Different Lag Weeks.

**Supplementary Table 3.** Subgroup Heterogeneity Test of Meteorological Factors.

**Supplementary Table 4.** Basic Characteristics of Included Studies.

**Supplementary Table 5.** Subgroup analysis of the impact of temperature on influenza risk.

**Supplementary Table 6.** Subgroup analysis of the impact of relative humidity on influenza risk.

**Supplementary Table 7.** Meta-regression analysis of influenza on temperature and relative humidity.

**Supplementary Table 8.** Publication bias test of temperature and relative humidity.

# Supplementary Figures

**Supplementary Figure 1.** Time distribution of weekly influenza and meteorological factors in Wuxi city, 2014-2019.

**Supplementary Figure 2.** Systematic search and study selection.

**Supplementary Figure 3.** Funnel plot of the association between temperature, relative humidity and influenza.

# References

This systematic review and meta-analysis were conducted following the Preferred Reporting Items for Systematic Reviews and Meta-Analyses (PRISMA) guidelines (1). The study has been registered with PROSPERO under the registration number CRD42021284952.

# Search strategy (Taking PubMed as an example)

#1 "Influenza, Human"[Mesh]

#2 Influenza[Title/Abstract]

#3 #1 OR #2

#4 ambient temperature[Title/Abstract] OR temperature[Title/Abstract]

#5 relative humidity[Title/Abstract] OR humidity[Title/Abstract]

#6 rainfall[Title/Abstract]) OR precipitation[Title/Abstract]

#7 air pressure[Title/Abstract]) OR atmospheric pressure[Title/Abstract]

#8 wind speed[Title/Abstract] OR wind velocity[Title/Abstract]

#9 sunshine[Title/Abstract]) OR sunshine duration[Title/Abstract]

#10 meteorology[Title/Abstract] OR climate[Title/Abstract]

#11 #4 OR #5 OR #6 OR #6 OR #8 OR #9 OR #10

#12 #3 AND #11

# Quality assessment

The quality of the included studies was assessed using the research quality evaluation criteria recommended by the Agency for Healthcare Research and Quality (AHRQ), which consists of 11 items (2). A score of 0 to 3 indicates low quality, 4 to 7 indicates medium quality, and 8 to 11 indicates high quality. The results are presented in Supplementary Table 4.

# Study characteristics

Initially, 14,096 articles were identified. After removing duplicates and applying inclusion/exclusion criteria, 13 studies were selected. A data update in January 2025 added one more study, bringing the total to 14 studies for meta-analysis. The screening process is shown in Supplementary Figure 2. Of these studies, 13 focused on temperature, 4 on relative humidity, 2 on precipitation, 1 on sunshine duration, and 1 on wind speed. The studies included 10 from subtropical regions, 2 from temperate regions, and 1 from tropical regions. Two studies targeted children, and 12 focused on the general population, with 2 including age stratification. Five studies analyzed different influenza subtypes. The studies were summarized for meta-analysis, with temperature effects categorized into cold, hot, and average to assess their role in influenza incidence. Subgroup analysis was performed based on population and regional climate to identify sources of heterogeneity.

# Supplementary Tables

**Supplementary Table 1.** Spearman correlation between influenza cases and meteorological factors in Wuxi, 2014-2019.

| **Correlation** | **Cases** | **Pressure** | **Temperature** | **Relative humidity** | **Precipitation** | **Wind speed** | **Sunshine duration** |
| --- | --- | --- | --- | --- | --- | --- | --- |
| **Cases** | 1 |  |  |  |  |  |  |
| **Pressure** | 0.478 ^*^ | 1 |  |  |  |  |  |
| **Temperature** | -0.539 ^*^ | -0.933 ^*^ | 1 |  |  |  |  |
| **Relative humidity** | -0.005 | -0.150 ^*^ | 0.103 | 1 |  |  |  |
| **Precipitation** | -0.040 | -0.297 ^*^ | 0.183 ^*^ | 0.617 ^*^ | 1 |  |  |
| **Wind speed** | -0.036 | -0.221 ^*^ | 0.137 ^*^ | -0.095 | 0.167 ^*^ | 1 |  |
| **Sunshine duration** | -0.117 ^*^ | -0.093 | 0.188 ^*^ | -0.754 ^*^ | -0.542 ^*^ | 0.066 | 1 |

^*^ P < 0.05

**Supplementary Table 2.** GCV Scores of Meteorological Factors and Influenza Incidence Risk Models for Different Lag Weeks.

| **Lag weeks** | **Pressure** | **Temperature** | **Relative humidity** | **Precipitation** | **Wind speed** | **Sunshine duration** |
| --- | --- | --- | --- | --- | --- | --- |
| **Lag0** | 11.12556 | 11.22518 | 10.29512 | 11.30741 | 11.68140 | 10.67005 |
| **Lag1** | 11.38494 | 11.45404 | 11.74052 | 11.75474 | 11.71740 | 11.71375 |
| **Lag2** | 10.61353 | 10.23179 | 11.60400 | 11.72039 | 11.30681 | 11.65771 |

**Supplementary Table 3.** Subgroup Heterogeneity Test of Meteorological Factors.

| Meteorological variables | Sex | Age |
| --- | --- | --- |
| Pressure | Q=0.02, *P*=0.8775 | Q=11.32, *P*=0.0101 ^*^ |
| Temperature | Q=0.00, *P*=0.9475 | Q=10.14, *P*=0.0174 ^*^ |
| Wind speed | Q=0.00, *P*=0.9616 | Q=1.37, *P*=0.7127 |
| Relative humidity | Q=0.03, *P*=0.8736 | Q=3.15, *P*=0.3687 |
| Precipitation | Q=0.27, *P*=0.6015 | Q=29.73, *P*<0.0001 ^*^ |
| Sunshine duration | Q=0.00, *P*=0.9826 | Q=17.46, *P*=0.0006 ^*^ |

^*^ P < 0.05

**Supplementary Table 4.** Basic Characteristics of Included Studies.

| First author  (year) | Published | Period | Location | Population | Age | Exposure | Study design | Statistical model | Climate group | Outcome | Quality scores |
| --- | --- | --- | --- | --- | --- | --- | --- | --- | --- | --- | --- |
| Liu et al (3) | 2019 | 2012-2017 | Wuxi,China | 1573 | All | T | Time series study | DLNM | Subtropical | Confirmed Cases | 6 |
| Chen et al (4) | 2021 | 2004-2019 | China | 1986536 | ≤14 year  15-59 year  ≥60 year | T | Time series study | GAM | Subtropical and temperate | Confirmed Cases | 5 |
| Jaakkola et al (5) | 2014 | 2004-2015 | Finland | 66 | All | T | Case-crossover study | Conditional logistic regression | Subtropical | Confirmed Cases | 9 |
| Lau et al (6) | 2020 | 2014-2018 | Hangzhou, China | 20165 | All | PRCP、T、RH | Time series study | GAM, DLNM | Subtropical | Confirmed Cases | 7 |
| Li et al (7) | 2022 | 2014-2017 | Wuhan, China | 12390 | All | T | Time series study | GAM, DLNM | Subtropical | Confirmed Cases | 6 |
| Li et al (8) | 2020 | 2017-2019 | Shanghai, China | 29315 | 0-18 years | T | Time series study | DLNM | Subtropical | Confirmed Cases | 7 |
| Ma et al (9) | 2022 | 2009-2015 | Shenzhen, China | 3330 | All | T、RH、WS | Time series study | GAM, DLNM | Subtropical | Confirmed Cases | 7 |
| Ng et al (10) | 20222022 | 2014-2018 | Macau, China | 17104 | All | T、RH、SD | Time series study | DLNM | Subtropical | Confirmed Cases | 7 |
| Wang et al (11) | 2022 | 2010-2019 | Lanzhou, China | 6701 | All | RH、T | Time series study | DLNM | Temperate | Confirmed Cases | 6 |
| Wu et al (12) | 2021 | 2014-2019 | Hefei, China | 5238 | 0-4 year  5-17 year  ≥18 year | T | Time series study | GAM, DLNM | Subtropical | Confirmed Cases | 7 |
| Xu et al (13) | 2013 | 2010-2018 | Brisbane, Australia | 2922 | 0-14 years | T | Time series study | Poisson log-linear regression | Subtropical | Confirmed Cases | 5 |
| Soebiyanto et al (14) | 2014 | 2008-2013 | Guatemala、El Salvador and Panama | 6420 | All | T、PRCP | Time series study | Logistic regression | Tropical | Confirmed Cases | 6 |
| Wafatsuma et al (15) | 2024 | 2014.3-2019.12 | Kawasaki, Japan | 181895 | All | T | Time series study | GLM、DLNM | Temperate | Confirmed Cases | 7 |

Abbreviations: DLNM, Distributed Lag Non-Linear Model; GAM, Generalized Additive Model; GLM, Generalized Linear Model; NA, Not Available; PRCP, Precipitation; RH, Relative humidity; SD, Sunshine duration; T, Temperature; WS, Wind speed.

**Supplementary Table 5.** Subgroup analysis of the impact of temperature on influenza risk.

| Subgroup types | Cold effect | | |  | Hot effect | | |
| --- | --- | --- | --- | --- | --- | --- | --- |
|  | n | Pooled RR(95%CI) | *I^2^* %, *P* value |  | n | Pooled RR(95%CI) | *I^2^* %, *P* value |
| Population |  |  |  |  |  |  |  |
| All | 8 | 0.9193(0.8900~0.9496) ^*^ | 87.4%,0.0001 |  | 9 | 1.0595(1.0148~1.1061) ^*^ | 81.7%, <0.0001 |
| Children only | 2 | 0.7291(0.6289~0.8453) ^*^ | 91.0%, 0.0008 |  | 1 | 1.2693(1.2071~1.3348) ^*^ | NA |
| Regional climate |  |  |  |  |  |  |  |
| Subtropical | 7 | 0.8658(0.7858~0.9540) ^*^ | 96.6%, 0.0001 |  | 8 | 1.1035(1.0300~1.1822) ^*^ | 93.2%, <0.0001 |
| Temperate | 3 | 0.8977(0.8593~0.9378) ^*^ | 80.8%, *P*=0.0054 |  | 2 | 1.0567(0.8484~1.3162) | 91.4%, 0.0007 |

^*^ P < 0.05

**Supplementary Table 6.** Subgroup analysis of the impact of relative humidity on influenza risk.

| Subgroup types | Low effect | | |  | High effect | | |
| --- | --- | --- | --- | --- | --- | --- | --- |
|  | n | Pooled RR(95%CI) | *I^2^* %, *P* value |  | n | Pooled RR(95%CI) | *I^2^* %, *P* value |
| Population |  |  |  |  |  |  |  |
| All | 8 | 0.9603(0.9400~0.9811) ^*^ | 96.1%, *P*<0.0001 |  | 6 | 1.0507(1.0137~1.0890) ^*^ | 87.5%, 0.0001 |
| Children only | 4 | 0.9646(0.9553~0.9740) ^*^ | 0.00%, *P*=0.6489 |  | 4 | 1.0508(1.0299~1.0721) ^*^ | 52.8%, 0.0953 |
| Regional climate |  |  |  |  |  |  |  |
| Subtropical | 11 | 0.9684(0.9549~0.9821) ^*^ | 90.3%, *P*<0.0001 |  | 10 | 1.0492(1.0276~1.0711) ^*^ | 84.7%, 0.0001 |
| Temperate | 1 | 0.9238(0.9122~0.9353) ^*^ | NA |  | 0 |  |  |

^*^ P < 0.05

**Supplementary Table 7.** Meta-regression analysis of influenza on temperature and relative humidity.

| Index | Effect | Factors | Estimate | se | zval | P value | ci.lb | ci.ub |
| --- | --- | --- | --- | --- | --- | --- | --- | --- |
| Temperature | Cold effect | Population | -0.2374 | 0.0468 | -5.0748 | <0.0001 ^*^ | -0.3290 | -0.1457 |
|  |  | Regional climate | -0.0410 | 0.0380 | -1.0567 | 0.2906 | -0.1145 | 0.0343 |
|  | Hot effect | Population | 0.1715 | 0.0806 | 2.1263 | 0.0335 ^*^ | 0.0134 | 0.3295 |
|  |  | Regional climate | -0.0140 | 0.0669 | -0.2095 | 0.8341 | -0.1451 | 0.1170 |
| Relative humidity | Low relative humidity | Population | 0.0001 | 0.0162 | 0.0066 | 0.9947 | -0.0316 | 0.0318 |
|  |  | Regional climate | -0.0469 | 0.0237 | -1.9787 | 0.0479 ^*^ | -0.0933 | -0.0004 |
|  | High relative humidity | Population | 0.0032 | 0.0230 | 0.1383 | 0.8900 | -0.0420 | 0.0483 |

^*^ P < 0.05

**Supplementary Table 8.** Publication bias test of temperature and relative humidity.

| Variable | Effect | n | Method | Statistics | *P* value |
| --- | --- | --- | --- | --- | --- |
| Ambient temperature | Cold effect | 10 | Egger’s test | -2.70 | 0.0270 |
|  |  |  | Begg’s test | -1.34 | 0.1797 |
|  | Hot effect | 10 | Egger’s test | 2.50 | 0.0369 |
|  |  |  | Begg’s test | 0.80 | 0.4208 |
|  | Mean effect | 5 ^*^ | Egger’s test | -1.53 | 0.2236 |
|  |  |  | Begg’s test | -0.49 | 0.6242 |
| Relative humidity | Low effect | 8 ^*^ | Egger’s test | -2.43 | 0.0513 |
|  |  |  | Begg’s test | 0.00 | 1.0000 |
|  | High effect | 6 ^*^ | Egger’s test | 2.38 | 0.0756 |
|  |  |  | Begg’s test | 1.69 | 0.0909 |

^*^ Since Egger’s and Begg’s tests are recommended to be conducted with more than 10 studies, the results for some groups that include fewer studies may be biased.

# Supplementary Figures


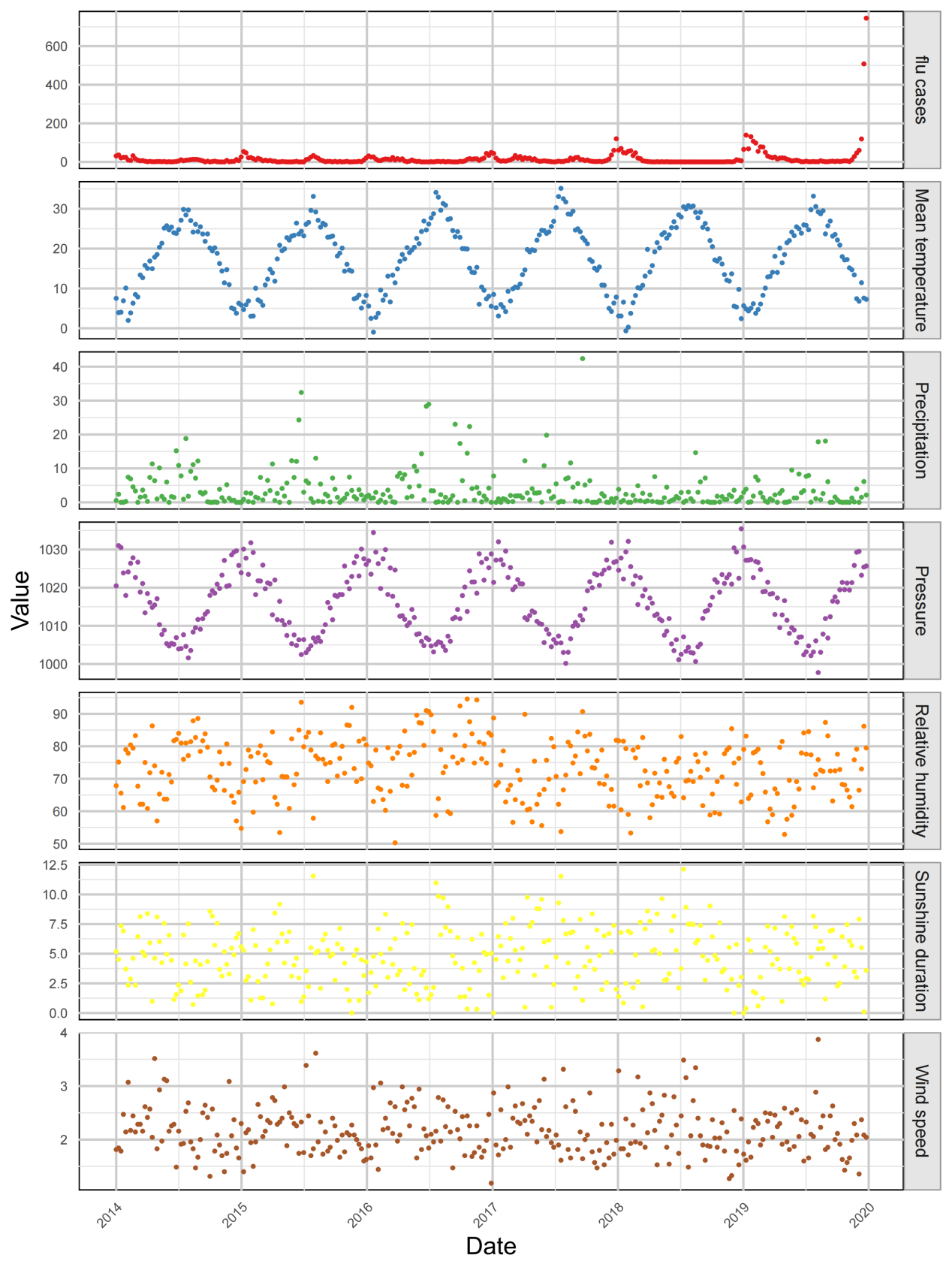


**Supplementary Figure 1.** Time distribution of weekly influenza and meteorological factors in Wuxi city, 2014-2019.


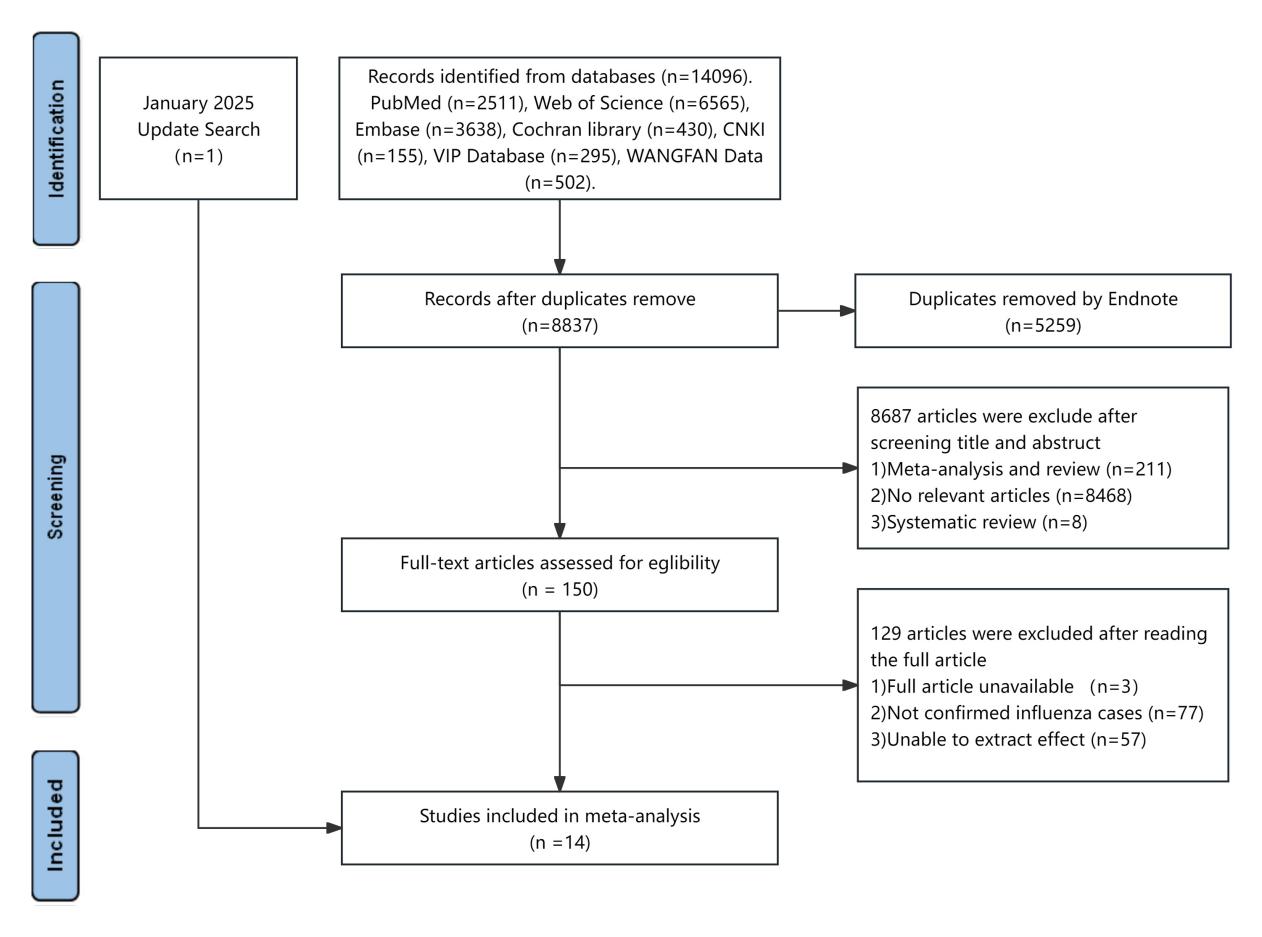


**Supplementary Figure 2.** Systematic search and study selection.

**A**

**B**


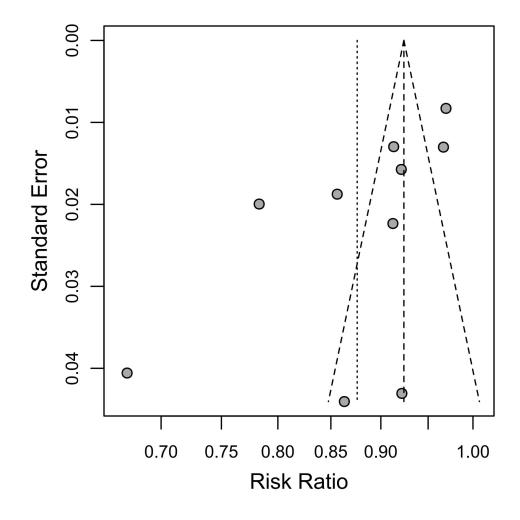

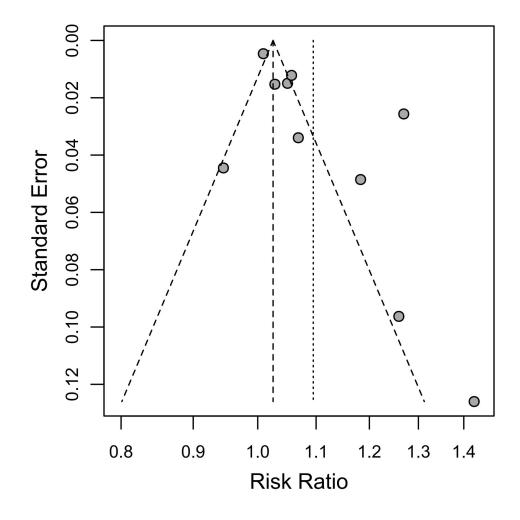


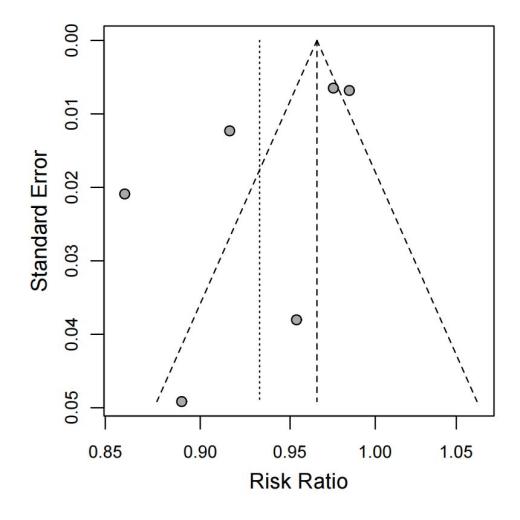


**C**


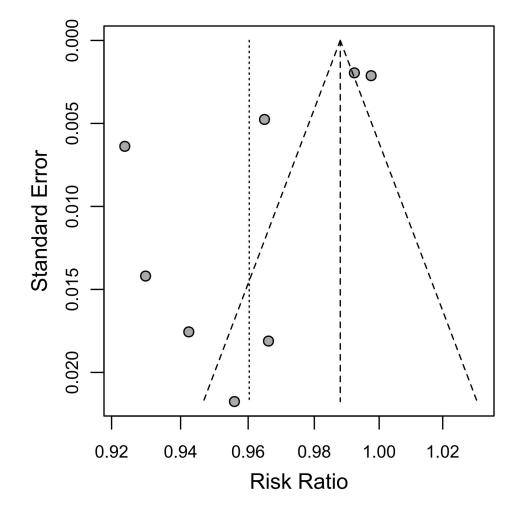

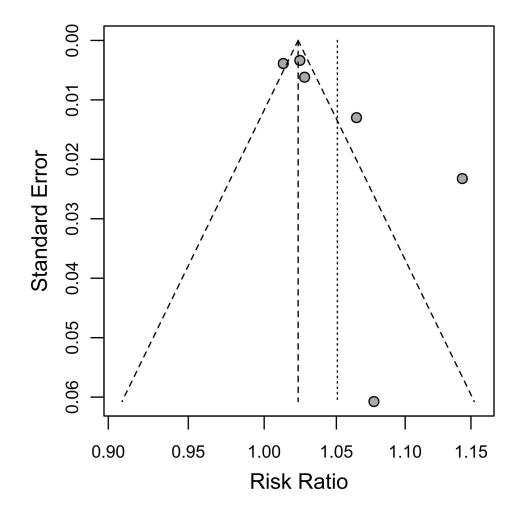


**E**

**D**

**Supplementary Figure S3.** Funnel plot of the association between temperature, relative humidity and influenza. **A** for cold effect of temperature, **B** for hot effect of temperature, **C** for average effect of temperature, **D** for dry effect of relative humidity, **E** for wet effect of relative humidity.

# References:

1. Moher D, Liberati A, Tetzlaff J, Altman DG. Preferred Reporting Items for Systematic Reviews and Meta-Analyses: The Prisma Statement. *PLoS Med* (2009) 6(7):e1000097. Epub 2009/07/22. doi: <http://doi.org/10.1371/journal.pmed.1000097>.

2. Huang T, Li C, Chen F, Xie D, Yang C, Chen Y, et al. Prevalence and Risk Factors of Osteosarcopenia: A Systematic Review and Meta-Analysis. *BMC Geriatr* (2023) 23(1):369. Epub 2023/06/16. doi: <http://doi.org/10.1186/s12877-023-04085-9>.

3. Liu J, Shi C, Zhang Q, Shi P. The Effect of Ambient Temperature on the Activity Influenza Like Illness Andlaboratory-Confirmed Influenza in Wuxi City. *Chin J Dis Control Prev* (2019) 23(11):1389-93+419. doi: <http://doi.org/10.16462/j.cnki.zhjbkz.2019.2019.11.018>.

4. Chen C, Zhang X, Jiang D, Yan D, Guan Z, Zhou Y, et al. Associations between Temperature and Influenza Activity: A National Time Series Study in China. *Int J Environ Res Public Health* (2021) 18(20):10846. Epub 2021/10/24. doi: <http://doi.org/10.3390/ijerph182010846>.

5. Jaakkola K, Saukkoriipi A, Jokelainen J, Juvonen R, Kauppila J, Vainio O, et al. Decline in Temperature and Humidity Increases the Occurrence of Influenza in Cold Climate. *Environ Health* (2014) 13(1):22. Epub 2014/04/01. doi: <http://doi.org/10.1186/1476-069x-13-22>.

6. Lau SY, Cheng W, Yu Z, Mohammad KN, Wang MH, Zee BC, et al. Independent Association between Meteorological Factors, Pm2.5, and Seasonal Influenza Activity in Hangzhou, Zhejiang Province, China. *Influenza Other Respir Viruses* (2021) 15(4):513-20. Epub 2020/12/21. doi: <http://doi.org/10.1111/irv.12829>.

7. Li Y, Wu J, Hao J, Dou Q, Xiang H, Liu S. Short-Term Impact of Ambient Temperature on the Incidence of Influenza in Wuhan, China. *Environ Sci Pollut Res Int* (2022) 29(12):18116-25. Epub 2021/10/23. doi: <http://doi.org/10.1007/s11356-021-16948-y>.

8. Li Y, Ye X, Zhou J, Zhai F, Chen J. The Association between the Seasonality of Pediatric Pandemic Influenza Virus Outbreak and Ambient Meteorological Factors in Shanghai. *Environmental Health* (2020) 19(1). doi: <http://doi.org/10.1186/s12940-020-00625-7>.

9. Ma P, Tang X, Zhang L, Wang X, Wang W, Zhang X, et al. Influenza a and B Outbreaks Differed in Their Associations with Climate Conditions in Shenzhen, China. *International Journal of Biometeorology* (2021) 66(1):163-73. doi: <http://doi.org/10.1007/s00484-021-02204-y>.

10. Ng H, Li Y, Zhang T, Lu Y, Wong C, Ni J, et al. Association between Multiple Meteorological Variables and Seasonal Influenza a and B Virus Transmission in Macau. *Heliyon* (2022) 8(11):e11820. Epub 2022/12/06. doi: <http://doi.org/10.1016/j.heliyon.2022.e11820>.

11. Wang J, Zhang L, Lei R, Li P, Li S. Effects and Interaction of Meteorological Parameters on Influenza Incidence During 2010-2019 in Lanzhou, China. *Front Public Health* (2022) 10:833710. Epub 2022/03/12. doi: <http://doi.org/10.3389/fpubh.2022.833710>.

12. Wu Q, He J, Zhang W-Y, Zhao K-F, Jin J, Yu J-L, et al. The Contrasting Relationships of Relative Humidity with Influenza a and B in a Humid Subtropical Region. *Environmental Science and Pollution Research* (2021) 28(27):36828-36. doi: <http://doi.org/10.1007/s11356-021-13107-1>.

13. Xu Z, Hu W, Williams G, Clements ACA, Kan H, Tong S. Air Pollution, Temperature and Pediatric Influenza in Brisbane, Australia. *Environment International* (2013) 59:384-8. doi: <http://doi.org/10.1016/j.envint.2013.06.022>.

14. Soebiyanto RP, Clara W, Jara J, Castillo L, Sorto OR, Marinero S, et al. The Role of Temperature and Humidity on Seasonal Influenza in Tropical Areas: Guatemala, El Salvador and Panama, 2008-2013. *PLoS One* (2014) 9(6):e100659. Epub 2014/06/24. doi: <http://doi.org/10.1371/journal.pone.0100659>.

15. Wagatsuma K. Effect of Short-Term Ambient Temperature Exposure on Influenza a and B Incidence: A Time-Series Analysis of Daily Surveillance Data in Kawasaki City, Japan. *IJID Reg* (2024) 13:100479. Epub 2024/12/03. doi: <http://doi.org/10.1016/j.ijregi.2024.100479>.
